# Star-shaped tetrathiafulvalene oligomers towards the construction of conducting supramolecular assembly

**DOI:** 10.3762/bjoc.11.175

**Published:** 2015-09-10

**Authors:** Masahiko Iyoda, Masashi Hasegawa

**Affiliations:** 1 Department of Chemistry, Graduate School of Science and Engineering, Tokyo Metropolitan University, Hachioji, Tokyo 192-0397, Japan; 2 Department of Chemistry, School of Science, Kitasato University, 1-15-1 Kitasato, Minami-ku, Sagamihara, Kanagawa 252-0373, Japan

**Keywords:** conducting fibers, star-shaped molecules, supramolecular assembly, tetrathiafulvalene oligomers

## Abstract

The construction of redox-active supramolecular assemblies based on star-shaped and radially expanded tetrathiafulvalene (TTF) oligomers with divergent and extended conjugation is summarized. Star-shaped TTF oligomers easily self-aggregate with a nanophase separation to produce supramolecular structures, and their TTF units stack face-to-face to form columnar structures using the fastener effect. Based on redox-active self-organizing supramolecular structures, conducting nanoobjects are constructed by doping of TTF oligomers with oxidants after the formation of such nanostructures. Although radical cations derived from TTF oligomers strongly interact in solution to produce a mixed-valence dimer and π-dimer, it seems to be difficult to produce nanoobjects of radical cations different from those of neutral TTF oligomers. In some cases, however, radical cations form nanostructured fibers and rods by controlling the supramolecular assembly, oxidation states, and counter anions employed.

## Introduction

Tetrathiafulvalene (TTF) chemistry first attracted enthusiastic attention of chemists and physicists on high electrical conductivity and superconductivity with high *T*_c_ temperature. Recently, however, TTF and its derivatives are frequently employed as a redox-active moiety for organic electronic devices such as field-effect-transistors (FET), dye-sensitized solar cells (DSC), positive electrode materials for rechargeable batteries, and electrochromic (EC) materials [1].

TTF derivatives are versatile building blocks to form aggregates in the solid state with interesting conducting and magnetic behavior [2]. Although these properties are mainly originated from specific interactions between molecules having one or more unpaired electrons [3–4], neutral TTF and its derivatives also easily form stacked columnar structures with face-to-face π···π stacking and side-by-side S···S interactions in the crystalline state. Furthermore, weak intermolecular interactions (hydrogen bonding, metal coordination, CT interaction, π···π stacking, van der Waals interaction, etc.) play an important role in the formation of the three-dimensional (3D) crystal structures [5]. For the construction of nanostructured objects, π···π, S···S, and other weak intermolecular interactions first accelerate self-aggregation of molecules in solution [6–8] and then produce the functional one-dimensional (1D) or two-dimensional (2D) supramolecular structures, which are very important in advanced nanosciences [9–11]. For the formation of the nanostructured objects such as fibers, rods, tubes, and particles, amphiphilic TTFs having a rigid core and long alkyl chains are one of the best molecular systems. The self-assembly of TTF derivatives in solid and on surface gives rise to a long-distance dynamic ordering as compared with single crystals.

Among the recent researches on TTF and its derivatives, radially expanded or star-shaped multi-TTFs with *C*_3_ and *C*_6_ symmetries have attracted considerable attention in the field of materials science because of their divergent and extended π-conjugation. Various *C*_3_-symmetric compounds incorporating three conjugated TTF units were designed and synthesized to realize TTF-based conducting organic magnets using ferromagnetic interaction between the two TTF radical cations [12–21]. On the other hand, compounds with a hexagonal molecular symmetry were used as core structures for constructing conductive fibers and functional dyes [22–23]. Furthermore, various multifunctional TTF-based supramolecular architectures have been designed and synthesized to realize molecular sensors, redox switches, multi-input systems for logic gates, electrochemically-driven conformational controls, molecular clips and tweezers, and redox-controlled gelation processes. TTF-based supramolecular chemistry in solution was thoroughly outlined in recent reviews of Jeppesen, Nielsen, and Becher (2004) [24], Iyoda, Hasegawa, and Miyake (2004) [25–26], Sallé and Zhang (2009) [27], and Martin (2009, 2012) [28–29]. However, limited examples of redox-active nanostructures in the solid state were summarized so far. Therefore, this review focuses on the conducting nanostructures of TTF derivatives in the solid state, together with association behavior in solution.

## Review

### Redox-active radially expanded TTF oligomers in solution and the solid state

**Figure 1:**
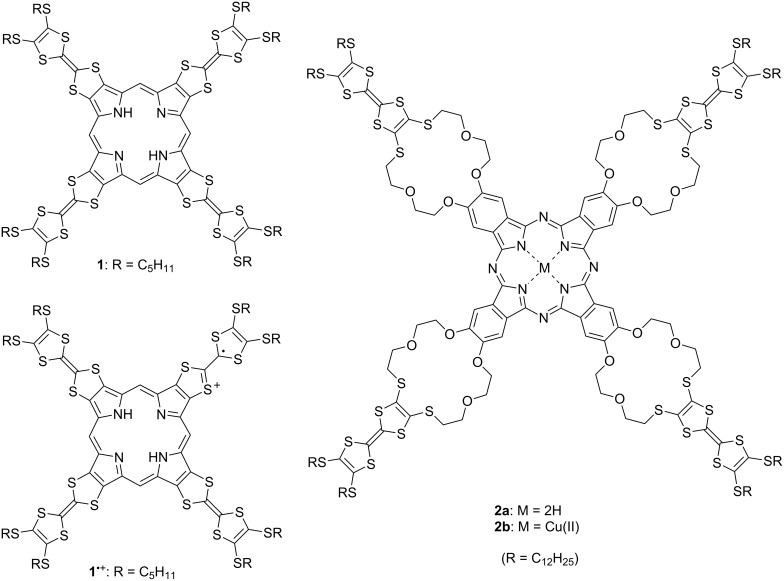
Radially expanded TTF oligomers **1** and **2a**,**b**.

TTF oligomers with radially expanded structures can be expected to demonstrate multifunctional properties because a central core and TTF branches exhibit individual and/or cooperative functionalities [25]. For example, dendrimers composed of central benzenoid cores and TTF branches are cited as representative examples [30–33]. Other functional units such as fullerenes [34–35], cyclodextrins [36], porphyrin [37], and phthalocyanine [38–40] can also be introduced into the core of radial oligo-TTFs. As shown in Figure 1, TTF-annelated porphyrin **1** was synthesized by Becher and co-workers in 2001 [37]. Reflecting its strong π-donor ability, **1** was oxidized spontaneously in solution to afford **1**^•+^ under ambient conditions. Multifunctional TTF-crown ether-substituted phthalocyanine (Pc) **2a** and its copper(II) complex **2b** were reported by Amabilino, Rowan, Nolte, and co-workers in 2005 [40]. The giant molecule **2a** self-aggregated in chloroform–dioxane to form a gel. TEM images of the xerogel exhibited helical molecular tapes nanometer wide and micrometer long. A cyclic voltammetry (CV) study on **2b** showed the redox properties expected for Pc and TTF, and doping of **2b** in CH_2_Cl_2_ with I_2_ produced a radical cation species.

**Figure 2:**
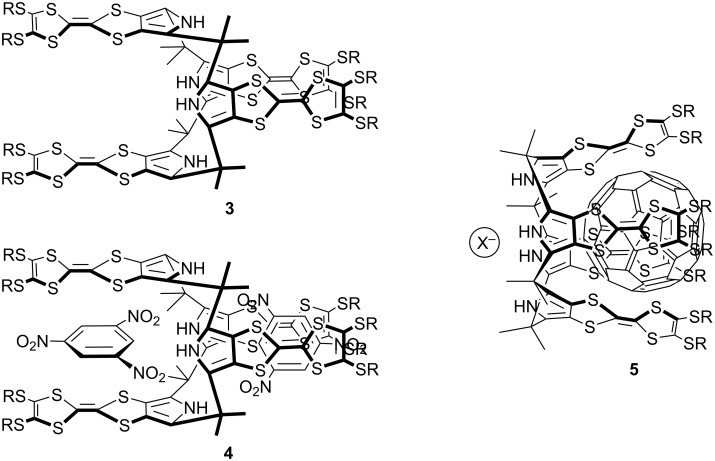
TTF-calix[4]pyrrole **3** and its TNT and C_60_ complexes **4** and **5**.

Among radially expanded TTFs, Jeppesen, Becher, Nielsen, Sessler, and co-workers reported TTF-calix[4]pyrrole **3** as a valuable supramolecular receptor, and **3** easily incorporated 1,3,5-trinitrobenzene (TNB) in the cavity to form **4** (**3**:TNB = 1:2) (Figure 2) [41–42]. Furthermore, in the presence of halide ions, **3** formed the C_60_ complex **5**, in which C_60_ was bound within the bowl-like cup of the TTF-calix[4]pyrrole core in a ball-and-socket binding mode [43].

**Figure 3:**
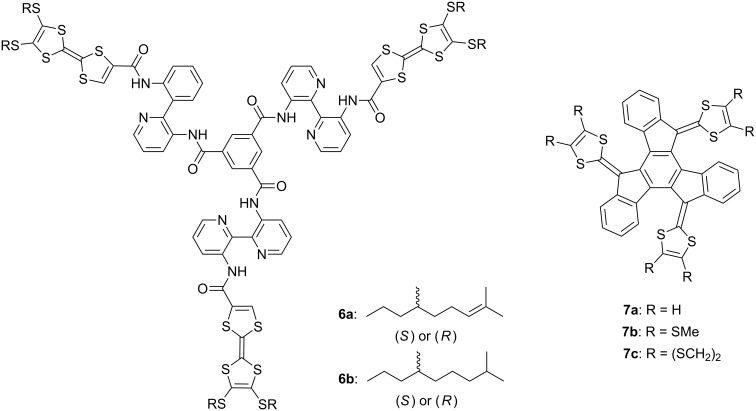
*C*_3_-symmetric TTF derivatives **6a,b** and **7a–c**.

Recently, the *C*_3_-symmetric compounds **6a**,**b** incorporating three TTF residues were reported by Amabilino, Avarvari, and co-workers (Figure 3) [21]. The three TTF units with chiral citronellyl and dihydrocitronellyl chains led to helical one-dimensional stacks in solution to produce fibers that have morphologies depending on the nature of the chiral alkyl group, although an achiral counterpart showed no helicity. *C*_3_-symmetric truxene-TTFs **7a–c** were synthesized by Ortí, Martín, and co-workers (Figure 3) [44].

**Figure 4:**
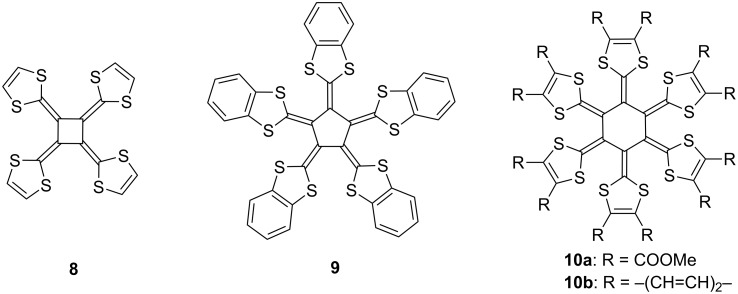
Radially expanded TTF derivatives **8**, **9**, and **10a**,**b**.

The pioneering studies on the synthesis of tetrakis(1,3-dithiol-2-ylidene)cyclobutane (**8**) and related [5] and [6]radialenes **9** and **10a**,**b** were reported by Yoshida and co-workers in the 1980’s (Figure 4). These π-expanded TTFs **8–10a**,**b** exhibited unique X-ray structures and multi-redox behavior [45–47].

### Conducting supramolecular assembly of oligo-TTFs

**Figure 5:**
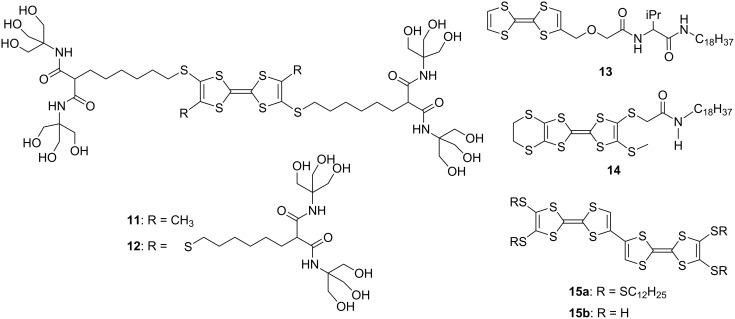
Amphiphilic TTFs **11–14** and **15a**,**b**.

The electric conductivities of doped nanofibers and nanorods derived from TTF and its derivatives are measured by mounting them on Au electrodes with a μm-sized spacing. On the other hand, the conductivities of the corresponding neutral nanoobjects are determined by pulse-radiolysis or flash-photolysis time-resolved microwave conductivity techniques [48–49]. Current-sensing atomic force microscopy (CS-AFM) and combination of scanning tunneling microscopy and spectroscopy (STM/STS) are also employed for determining the conductivities of nanoobjects [50–51]. The electrical conductivity of nanostructures mainly depends on the alignment of stacked TTFs or their radical salts. The first fibrous material was fabricated by using arborol-TTF **11** in 1994 by Joergensen, Bechgaard, and co-workers (Figure 5) [52]. Although **11** showed no conductivity, Bryce and co-workers synthesized arborol-functionalized TTF derivative **12** in 2003, whose doped film exhibited a moderate level of conductivity (σ_rt_ ≈ 10^−4^ S cm^−1^) [53]. In 2005, several groups reported the formation of nanofibers using amphiphilic TTFs (**13** and **14**) (Figure 5) [54–56] After that, many research efforts have been focused on the construction of conducting nanoobjects possibly employed as nanosized electric wires, wirings, molecular switches, and devices. Some neutral nanoobjects derived from TTFs show electroconductivity owing to the fastener effect [57]; however, the oxidation of face-to-face stacked TTFs easily generates highly conducting states with unfilled band structure. Thus, the doping of neutral nanoobjects with iodine is generally used for preparing conducting nanostructures. Chemically oxidized TTFs in solution are also available for preparing conducting nanofibers.

The fastener effect [57], which enhances the face-to-face interaction between the two TTFs, can be used to construct conducting nanostructured fibers in the neutral state, and doping of the fibers with iodine affords black conducting fibers. For example, the bi-TTF derivative **15a** with long alkylthio chains as substituents was synthesized (Figure 5) [58]. Bi-TTF **15a** formed reddish orange rods which exhibited a bulk conductivity of σ_rt_ = 1.0 × 10^−6^ S cm^−1^ without doping. The p-type semiconductivity was detected by CS-AFM. Furthermore, the doping of **15a** with iodine and bromine vapors afforded black conducting complexes (σ_rt_ = 1.1 × 10^−4^ and 1.5 × 10^−4^ S cm^−1^, respectively).

**Figure 6:**
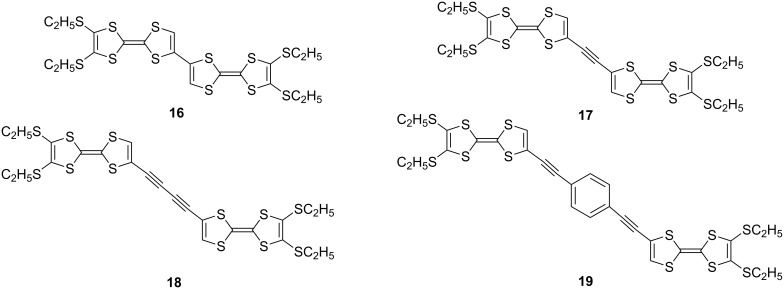
TTF dimers linked by σ-bond (**16**) and conjugated π-systems (**17–19**).

For conjugated TTF dimers linked by π-systems or chalcogen atoms, intramolecular through-bond and/or through-space interactions can be expected between two TTF parts. The intramolecular through-bond interaction between the two TTF parts linked in a head-to-tail manner is calculated to be weak in the ground state [25]. Thus, the conjugation of the two neutral TTF parts in **16–19** is weak (Figure 6) [59]. In the cyclic voltammetry (CV) measurements, tetraethylthio-bi-TTF **16** showed two one-electron and one two-electron redox waves (Table 1), while other TTF dimers of **17–19** exhibited only two two-electron reversible redox waves corresponding to TTF/TTF^•+^ and TTF^•+^/TTF^2+^ at a normal scan rate (100 mV s^-1^). As shown in Table 1, however, steady-state electronic spectra of **16**^•+^, **17**^•+^, and **18**^•+^ show intramolecular interaction between TTF and TTF^•+^, and the absorption maxima were observed at ca. 450 and 750 nm, together with broad absorption of intramolecular CT interactions between two TTF units at 1400, 1300, 1200 nm, respectively. The magnitude of these broad absorption bands is clearly affected by the distance between two TTFs, and TTF dimer **19**^•^**^+^** linked with a longer spacer exhibited no intramolecular CT absorption band. Moreover, the longest absorption maxima of the dications **16**^2+^, **17**^2+^, and **18**^2+^ exhibit a bathochromic shift of 44, 30, and 14 nm, respectively, from the corresponding absorption maximum of **16**^•+^, **17**^•+^, and **18**^•+^ due to the head-to-tail orientation of two TTF^•+^ (Davydov red shift) [25]. It is worth noting that the redox behavior of TTF dimers in CV measurements are sensitive to the concentration and the solvent used, and pristine bi-TTF **15b** showed two reversible two-electron redox waves at −0.03 and 0.38 V vs Fc/Fc^+^ in benzonitrile under normal conditions [25,60].

**Table 1:** Redox potentials of **16–19** and absorption maxima of monocations **16**^•+^, **17**^•+^, **18**^•+^ and **19**^•+^, and dications **16**^2+^, **17**^2+^, **18**^2+^ and **19**^2+^ [25,59].

Compound	Redox potentials^a^ vs Fc/Fc^+^			Absorption maxima^b^	
	*E*_1/2_^1^ (V)	*E*_1/2_^2^ (V)	*E*_1/2_^3^ (V)	Monocation (nm)	Dication (nm)

**16**	0.06	0.17	0.44	772, 1400 br	816, 1098 sh
**17**	0.11	0.42		778, 1300 br	808
**18**	0.12	0.42		790, 1200 br	804
**19**	0.08	0.38		790	796

^a^Potentials were measured by cyclic voltammetry (CV) in benzonitrile against a Ag/Ag^+^ electrode and adjusted to the Fc/Fc^+^ potential. ^b^Measured in CH_2_Cl_2_/CH_3_CN (4:1) using Fe(ClO_4_)_3_ as the oxidation reagent.

### Conducting nanostructure formation from star-shaped oligo-TTFs

**Scheme 1:**
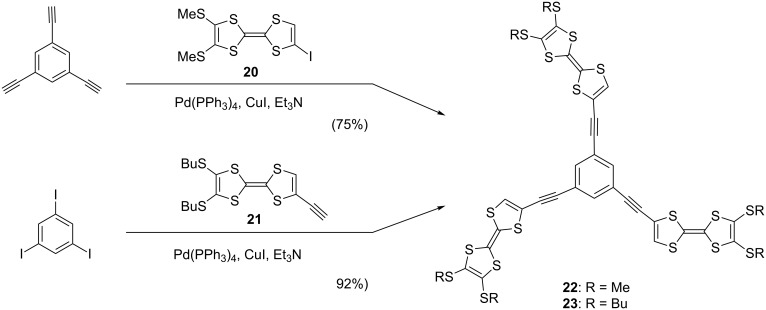
Synthesis of star-shaped TTF trimers **22** and **23**.

**Figure 7:**
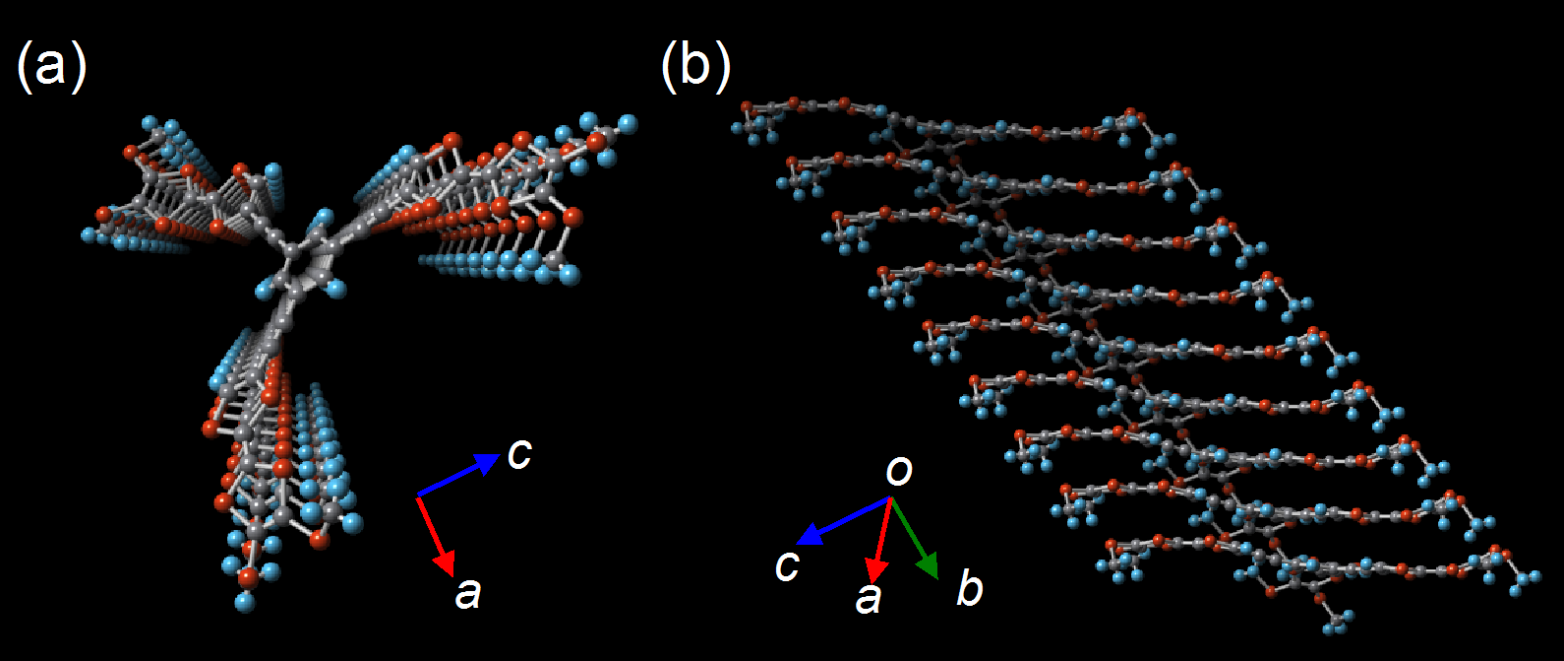
Projections of the molecular array of **22** in crystal structure (a) along with the *c* axis and (b) from side view.

Although pristine TTF does not self-associate in solution due to the low association constant for dimerization, the mixed-valence (MV) dyad (TTF/TTF)^•+^ and the dicationic dyad (TTF^•+^)_2_, so-called π-dimer, are formed in concentrated solution or at low temperature [61]. On the other hand, the synergy of either the fastener effect or π-expansion allows star-shaped *C*_3_-symmetric oligo-TTFs **22** and **23** to self-associate both in solution and in the solid state even in neutral state [18]. Compounds **22** and **23** were synthesized in good yields by Sonogashira coupling reaction of 1,3,5-triethynylbenzene with **20** and 1,3,5-triiodobenzene with **21**, respectively (Scheme 1). X-ray analysis of **22** revealed the columnar structure, in which the three TTF units stack in face-to-face manner to form single crystals (Figure 7).

In the case of **23** with butyl chains, this molecule dimerized in CDCl_3_ solution (*K*_2_ = 1.58 ± 0.30 M^−1^ at 293 K). The chemical shift of the central benzene ring clearly shifted higher field with an increase of concentration or lowering temperature. The observed shift is attributed to the shielding effect from the neighboring molecule that settles in face-to-face mode. The thermodynamic parameters were estimated to be Δ*H* = −9.43 kJ mol^−1^ and Δ*S* = −28.3 J mol^−1^ by the van’t Hoff plot (Table 2 and Supporting Information File 1). The self-association behavior is significantly affected by the solvent. While no association was observed in benzene-*d*_6_ solution in the concentration ranges of 0.7–21 mM even at low temperatures, a larger *K*_2_ value was estimated in CDCl_3_–CD_3_CN solution (3:7 v/v, *K*_2_ = 5.01 ± 0.98 M^−1^ at 293 K). Moreover, only a small concentration dependence of the chemical shift, which could not be used for determination of the *K*_2_ value, was observed in acetone–CS_2_ solution owing to very weak self-association. These results clearly suggest that the association behavior is driven by intermolecular π–π, S···S, and/or S···H interactions in solution. Note that these *K*_2_ values of **23** in the neutral state are similar to that of the mixed valence dimer (TTF^•+^ + TTF) (*K*_2_ = 6.0 M^−1^) and much larger than that of the π-dimer (TTF^•+^ + TTF^•+^) (*K*_2_ = 0.6 M^−1^) described in the literature [61].

**Table 2:** Association (dimerization) constants and thermodynamic parameters of **23** in various solvents^a^.

Solvent	*K*_2_ (M^−1^)^b^	Δ*G* (kJ mol^−1^)^b^	Δ*H* (kJ mol^−1^)	Δ*S* (J mol^−1^)

CDCl_3_	1.58	−1.13	−9.41	−28.2
CDCl_3_–CD_3_CN (3:7)	5.01	−3.64	−16.6	−43.1
benzene-*d*_6_	–^c^	–^c^	–^c^	–^c^

^a^Parameters were estimated from titration experiments using ^1^H NMR with the assumption of the dimerization process of **23**. ^b^At 298 K. ^c^No association was observed.

**Figure 8:**
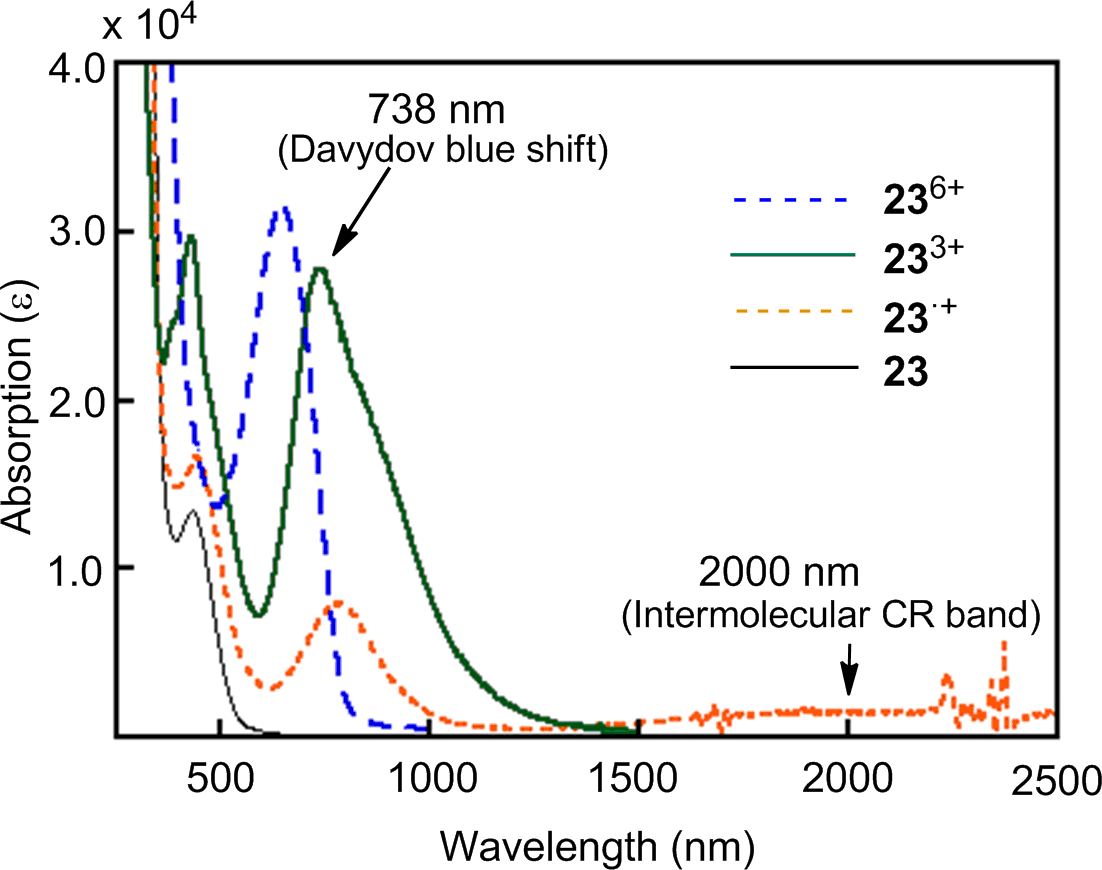
UV–vis/NIR spectra of **23**, **23**^•+^, **23**^3+^, and **23**^6+^.

Strong self-association of **23** was observed in the oxidation state. CV analysis of **23** in a dilute CH_2_Cl_2_ solution (1.9 × 10^−5^ M) showed two three-electron redox waves at 0.05 and 0.40 V vs Fc/Fc^+^ corresponding to the formation of **23**^3+^ and **23**^6+^, whereas a similar CV analysis of **23** in a concentrated CH_2_Cl_2_ solution (1.2 × 10^−3^ M) displayed three reversible waves at −0.04, 0.14, and 0.47 V vs Fc/Fc^+^ corresponding to the formation of (**23**)_2_^3+^, (**23**)_2_^6+^, and (**23**)_2_^12+^ (Figure S2, Supporting Information File 1). Interestingly, the three cationic species **23**^•+^, **23**^2+^ and **23**^3+^ prepared by chemical oxidation with Fe(ClO_4_)_3_ in CH_2_Cl_2_/CH_3_CN (4:1) showed a strong self-association, and electronic spectra of **23**^•+^ and **23**^2+^ exhibited marked intermolecular charge resonance (CR) bands at λ_max_ 2000 (br, ε 1500) and 2000 (br, ε 1700) owing to the face-to-face mixed valence interaction (Figure 8), and **23**^3+^ exhibited a typical Davydov blue shift (λ_max_ 738 nm, ε 27000) as compared with **19** (λ_max_ 796 nm, Table 1) [25]. To determine the conducting behavior of **22** and **23**, a pellet of **22** was treated with iodine to produce a semiconducting black solid (σ_rt_ = 3.6 × 10^−4^ S cm^−1^), whereas a similar doping of **23** with iodine resulted in the formation of the conducting liquid.

**Scheme 2:**
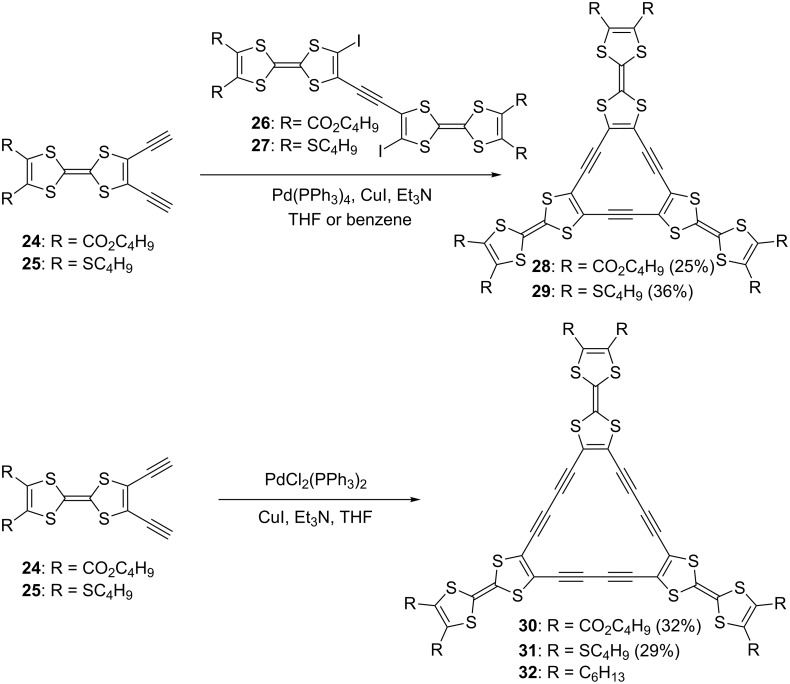
Synthesis of tris(TTF)[12]annulenes **28** and **29** and tris(TTF)[18]annulenes **30** and **31**, together with hexyl-substituted tris(TTF)[18]annulene **32**.

Radially expanded TTF oligomers with a large central π-surface can be expected to show effective intra- and intermolecular delocalization of electrons in the neutral and mixed-valence states. Furthermore, the supramolecular self-assembly of these large molecules having nanophase separation is a promising way of realizing molecular switches and devices [62–65]. With this in mind, hexadehydrotris(TTF)[12]annulenes **28** and **29** and dodecadehydrotris(TTF)[18]annulenes **30** and **31** were synthesized using palladium-mediated coupling reactions (Scheme 2) [20,25–26,66–68]. Tris(TTF)[12]annulenes **28** and **29** were pepared by Sonogashira coupling of **26** with **24** and **27** with **25** in 25 and 36% yields, respectively. For the synthesis of **30** and **31**, cyclotrimerization of **24** and **25** with a stoichiometric amount of PdCl_2_(PPh_3_)_2_ and CuI in triethylamine–THF was employed to afford **30** and **31** in 32 and 29% yields, respectively. Although tris(TTF)[18]annulenes are stable at room temperature in air, tris(TTF)[12]annulenes **28** and **29** gradually decomposed under ambient conditions due to the instability of central 4n π-electron system.

**Figure 9:**
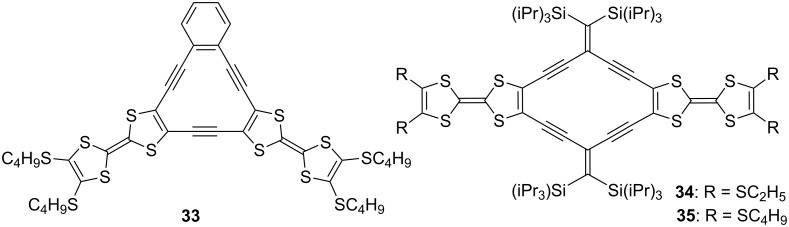
TTF-fused annulene **33** and radiannulenes **34** and **35**.

In order to investigate the effect of fused two TTF units on the cyclic conjugation and the interaction of the two TTF units in the neutral and cationic states, TTF-fused annulenes **33** [69] and radiannulenes **34** and **35** [70] were synthesized using a Sonogashira coupling in moderate yields (Figure 9).

**Figure 10:**
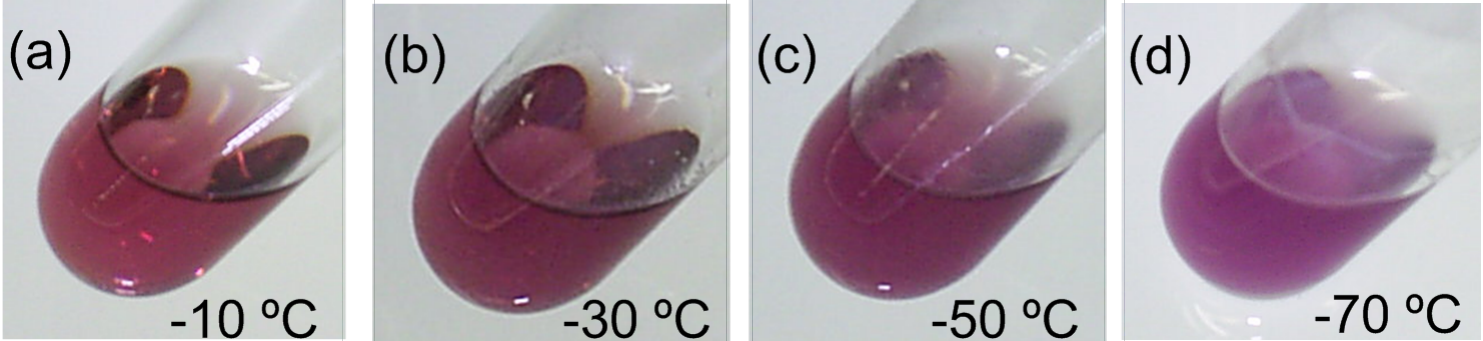
Colors of **30** solutions a–d in toluene (0.025 mM) at various temperatures. (a) λ_max_: 511 nm, (b) λ_max_: 512 nm, (c) λ_max_: 517 nm, (d) λ_max_: 520 nm. Reprinted with permission from [68]. Copyright 2012 Chemical Society of Japan.

**Figure 11:**
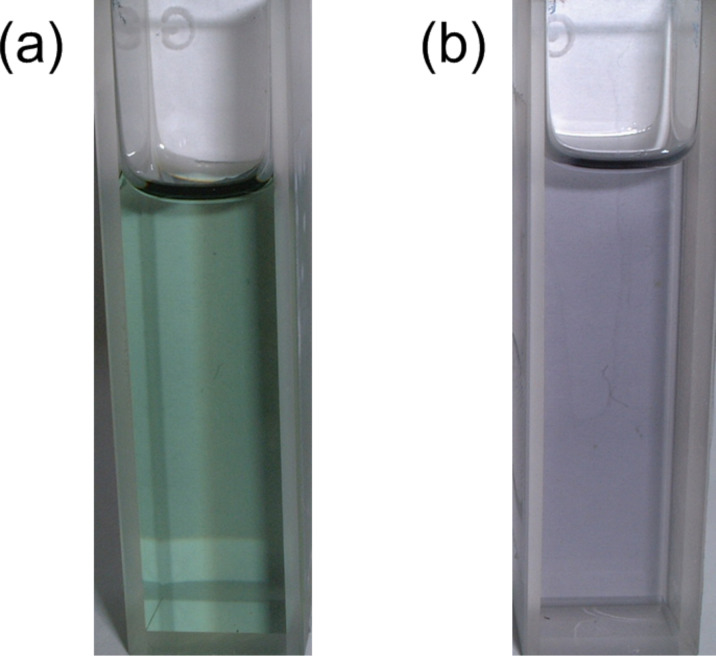
Solutions of **33**. (a) In CS_2_, λ_max_: 608 nm. (b) In CH_2_Cl_2_, λ_max_: 577 nm. Reprinted with permission from [69]. Copyright 2004 Royal Society of Chemistry.

The thermodynamic study on the self-aggregation of tris(TTF)annulenes indicates that the aggregation of **28**, **30**, and **31** is an enthalpically driven process that is entropically disfavored (Table 3) [68], although the aggregation of planar macrocyclic belts is both enthalpically and entropically driven [71]. The TTF[18]annulene **30** has smaller Δ*H* and Δ*S* values than the TTF[12]annulene **28**, suggesting a higher stacking ability and a larger ring size for **30**. Alkyl-substituted TTF[18]annulene **32** was reported to show almost no aggregation behavior in solution [72]. However, the slightly more amphiphilic **31** exhibits self-aggregation in benzene, toluene, and cyclohexane owing to a slightly larger nanophase separation in **31**. It is worth noting that the self-aggregation of TTF-annulenes results in the appearance of solvatochromism and thermochromism [68]. As shown in Figure 10, **30** exhibits a supramolecular thermochromism in toluene, and the color at −10 °C is reddish purple, whereas the color at −70 °C is purple. On the other hand, as shown in Figure 11, a solution of **33** exhibits deep green in CS_2_ but purple in CH_2_Cl_2_ [69].

**Table 3:** Self-aggregation data in toluene-*d*_8_.^a^

Comp.	Δ*G* (kJ mol^−1^) at 303 K	Δ*H* (kJ mol^−1^)	Δ*S* (J mol^−1^·K^−1^)

28	−11.8	−32.0	−66.3
30	−14.5	–37.8	−77.0
31	−10.1	−21.5	−37.1

^a^Determined with concentration/temperature-dependent ^1^H NMR assuming an infinite association model [68].

CV analysis of **28–34** in solution showed different behaviors (Table 4). The [12]annulenes **28**, **29**, and **33** exhibited two reversible one-electron reductions due to the smooth reduction of the 12π electron system to a 14π electron system, whereas the [18]annulenes **30** and **31** showed an irreversible reduction wave, due to the unfavorable reduction of the aromatic 18π electron system. On the other hand, all the molecules exhibited reversible oxidation waves in CH_2_Cl_2_ based on the high HOMO levels of TTF units. Another important feature in the CV data of **28–32** is broadening or splitting of the first oxidation wave, indicating intra- and/or intermolecular interactions between TTF units [68]. Interestingly, the first oxidation potential of **28** and **29** splited at the slow scanning rate of 3 mV s^−1^ owing to the intermolecular mixed-valence interaction between the TTF^•+^ and TTF moieties under diffusion-controlled conditions. In the case of **31**, the first oxidation potential (*E*_1/2_ = 0.14 V vs Fc/Fc^+^) is lower than that of **32** with alkyl groups (*E*_1/2_ = 0.20 V). Since the first oxidation potential of **31** in a dilute solution broadened but did not split (*E*_1/2_^ox1^ in CH_2_Cl_2_: 0.23 (3e) V), the potential of **31** at 0.14 V (Table 4) reflected the strong intermolecular interaction between the TTF**^•+^** and TTF moieties in **31**^•+^. By comparison with the known UV–vis/NIR spectra of mixed valence dimers [18,73], the association constant *K*_a_ of **31**^•+^ measured in CH_2_Cl_2_–MeCN 4:1 assuming an infinite association model [74] is large (*K*_a_ = 3.12 ± 0.48 × 10^5^ M^−1^ at 298 K) owing to 18 sulfur atoms in **31**. Therefore, the oxidation of **31** solution (0.1 mM) in CH_2_Cl_2_ first forms (**31**)_2_^2+^ owing to the intermolecular mix-valence interaction between the TTF**^•+^** and TTF moieties, and the further oxidation forms **31**^3+^ [68]. In summary, the oxidation of **28**–**31** showed multistep processes owing to intra- and/or intermolecular interactions between TTF units. In the case of [18]annulene **31**, the first oxidation potential splited in two with the strong intermolecular interaction in **31**^•+^. TTF-functionalized radiaannulenes (RAs) **34** and **35** also exhibit multiple redox states [70]. CV analysis of **34** shows the two reversible one-electron reductions as the reduction of the RA core, whereas the three reversible oxidations at 0.20, 0.29, and 0.61 V correspond to the formation of **34**^•+^, **34**^2+^, and **34**^4+^. Therefore, the redox behavior of **34** is similar to those of **28**, **29**, and **31**.

**Table 4:** Redox potentials of **28**–**34** measured by CV^a^.

Compound	*E*_1/2_^red2^ (V)	*E*_1/2_^red1^ (V)	*E*_1/2_^ox1^ (V)	*E*_1/2_^ox2^ (V)

**28**^b^	−1.52 (1e)	−1.16 (1e)	0.38 (3e) [0.29, 0.44]^c^	0.66 (3e)
**29**^b^	−1.78 (1e)	−1.41 (1e)	0.21 (3e) [0.12, 0.26]^c^	0.49 (3e)
**30**^b^	–^d^	–1.35^e^	0.43 (3e)^f^	0.70 (3e)
**31**^b^	–^d^	–1.48^e^	0.14 (1e), 0.29 (2e)	0.53 (3e)
**32**^g^	–^d^	–1.40^e^	0.20 (3e)	0.64 (3e)
**33**^b,i^	−1.87 (1e)	–1.50 (1e)	0.19 (2e)^f,h^	0.46 (2e)^h^
**34**^i^	−1.52 (1e)	−1.16 (1e)	0.20 (1e), 0.29 (1e)	0.61 (2e)

^a^Conditions: 0.1 M Bu_4_NClO_4_, 100 mV s^–1^, Pt as a working electrode, Ag/Ag^+^ as a reference electrode, Pt wire as a counter electrode. Potentials were referenced to Fc/Fc^+^. Solvent: THF for reduction, and CH_2_Cl_2_ for oxidation. ^b^Concentration: 0.1 mM. ^c^Measured at 3 mV s^–1^. ^d^Not observed. ^e^Irreversible process. ^f^Broad redox wave. ^g^According to [72]. ^h^Solvent: benzonitrile. ^i^According to [70].

**Figure 12:**
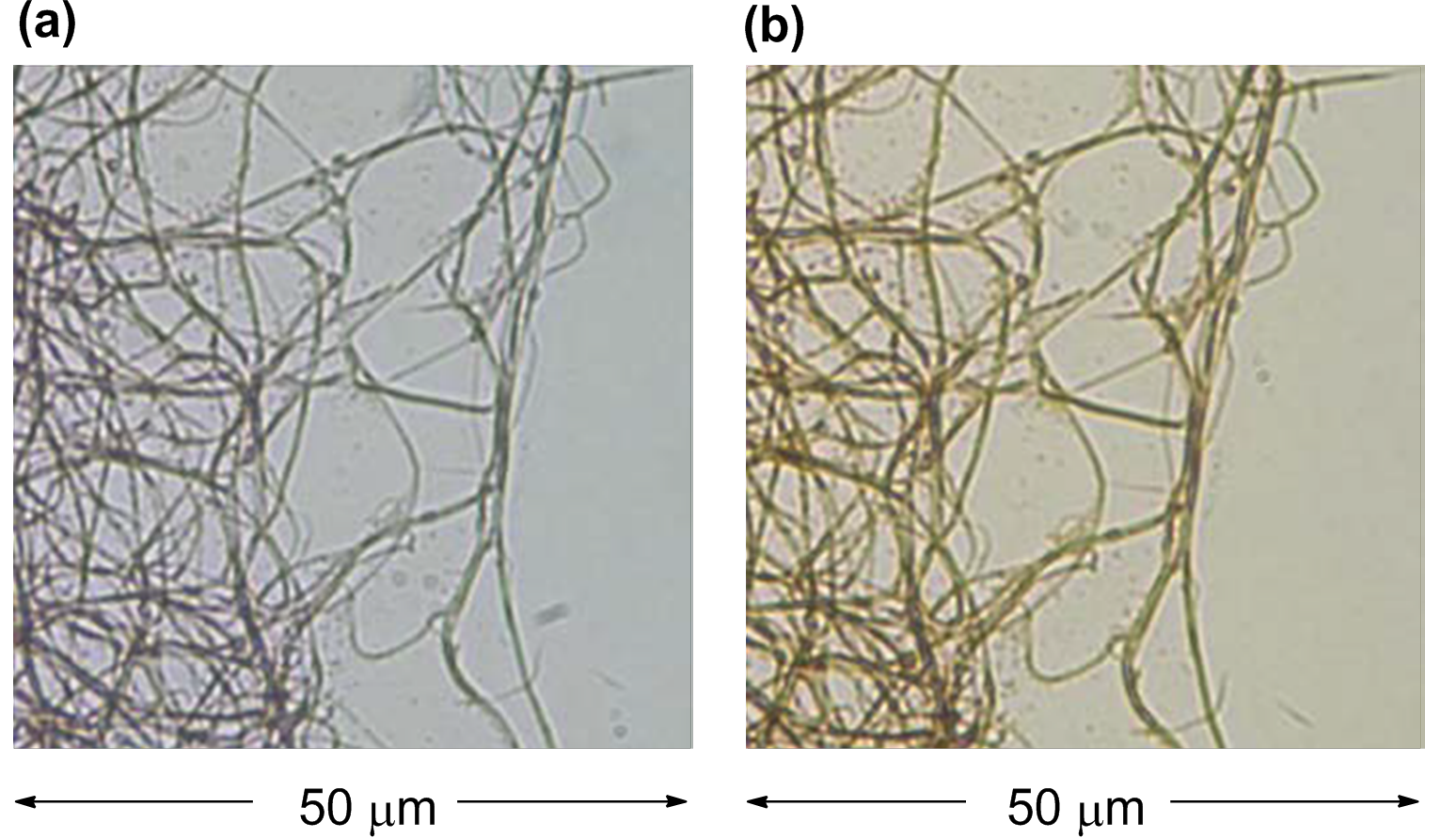
Optical micrographs (1000× magnified) of fibers, prepared from **30** in THF–H_2_O 1:1, on a glass plate at 23 °C. (a) Before iodine doping. (b) After iodine doping (3 min). Reprinted with permission from [26]. Copyright 2010 Royal Society of Chemistry.

The [18]annulenes **30** and **31** formed a fibrous structure in H_2_O–THF 1:1, and **31** required longer time for fiber formation than **30** owing to weaker association constant in solution (*K*_a_ in toluene-*d*_8_ at 303 K = 634 M^−1^ (**30**), 101 M^−1^ (**31**)) [67–68]. Both **30** and **31** fibers showed roughly the same behavior for doping with iodine, and the color of fibers quickly changed from bluish purple to dark brown due to the partial oxidation of **30** and **31** as shown Figure 12 (the maximum conductivities: **30** σ_rt_ 2.0 × 10^−2^ S cm^−1^, **31** σ_rt_ 2.6 × 10^−3^ S cm^−1^). The color of the doped fibers gradually returned to the original bluish purple under vacuum, but the speed of the iodine desorption for fiber **31** was very slow. The conductivity of the doped pellet prepared from fiber **30** is estimated to be ca. 1000 times higher than that of the neutral fiber (before doping: σ_rt_ 3 × 10−^6^ S cm^−1^, after doping: σ_rt_ 3 × 10^−3^ S cm^−1^) [68].

**Scheme 3:**
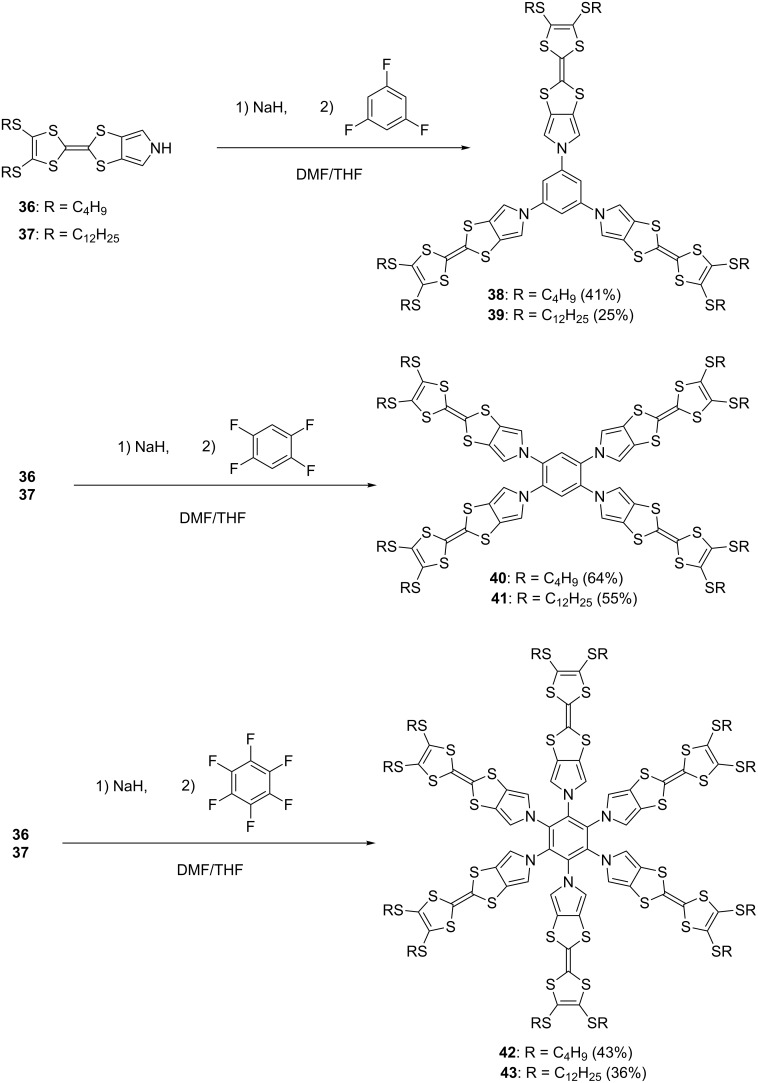
Star-shaped TTF oligomers **38–43**.

**Figure 13:**
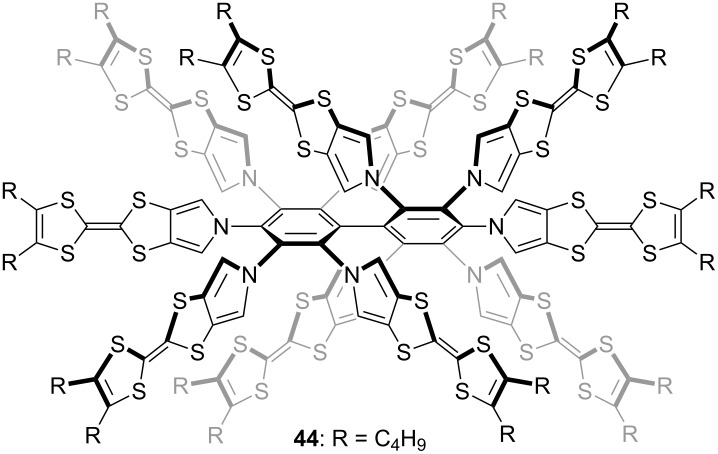
Star-shaped TTF 10-mer **44**.

Star-shaped pyrrole-fused TTF oligomers **38–43** were synthesized by nucleophilic aromatic substitution (S_N_Ar) reactions of fluorinated benzenes with the pyrrolyl sodium salts derived from **36** and **37** in moderate yields (Scheme 3) [23]. X-ray analysis of **38** revealed that the three TTF units are bent simply to fill an empty space and stacked to form a columnar structure. The torsion angle between the mean planes of the pyrrole and central benzene is 7–32°, indicating the conformational flexibility of the pyrrole–benzene linkage. The calculated torsion angles between the pyrroles and central benzenes of **38**, **40**, and **42** are 34, 45, and 59°, respectively, and the non-planar structures of **38**, **40**, and **42** are in good agreement with the high-field shift of α-protons of pyrroles in the ^1^H NMR spectra: δ 6.89 (**38**), 6.41 (**40**), 5.93 ppm (**42**). Star-shaped TTF 10-mer **44** was also synthesized by S_N_Ar reaction of the sodium salt of **36** with decafluorobiphenyl (44%) [75] (Figure 13).

**Figure 14:**
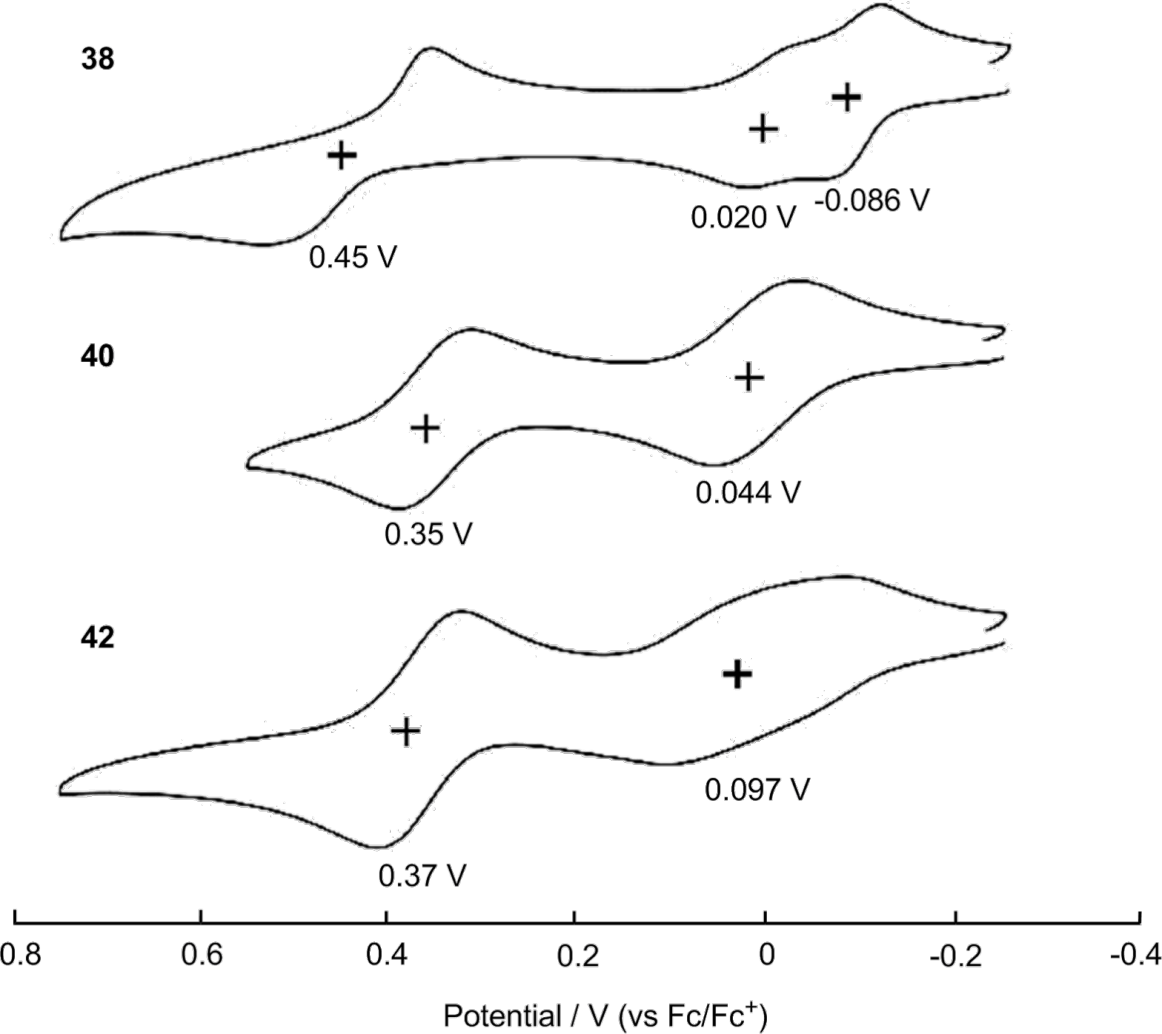
Cyclic voltammograms of **38**, **40**, and **42** (0.1 mM) in benzonitrile with 0.1 M *n*-Bu_4_PF_6_ as a supporting electrolyte, Ag/AgNO_3_ as a reference electrode, glassy carbon as a working electrode, Pt wire as a counter electrode, and a scan rate of 100 mV s^−1^. Values are half-wave potentials. Adapted with permission from [23]. Copyright 2011 American Chemical Society.

In the CV measurements (Figure 14), tetrasubstituted **40** shows typical two reversible oxidation waves at *E*_1/2_^ox1^ = 0.044 and *E*_1/2_^ox2^ = 0.35 V (vs Fc/Fc^+^). However, trisubstituted **38** and hexasubstituted **42** exhibit split and broad first peaks, respectively, at −0.086 and 0.020 V (**38**) and 0.097 V (**42**), followed by second peaks at 0.45 V (**38**) and 0.37 V (**42**). The CV data of tetrasubstituted **40** suggests no intramolecular charge delocalization between the adjacent TTF units. The splitting and broadening of the first oxidation waves in **38** and **42** are considered to be caused by intermolecular interactions between the neutral and cationic TTF units.

**Figure 15:**
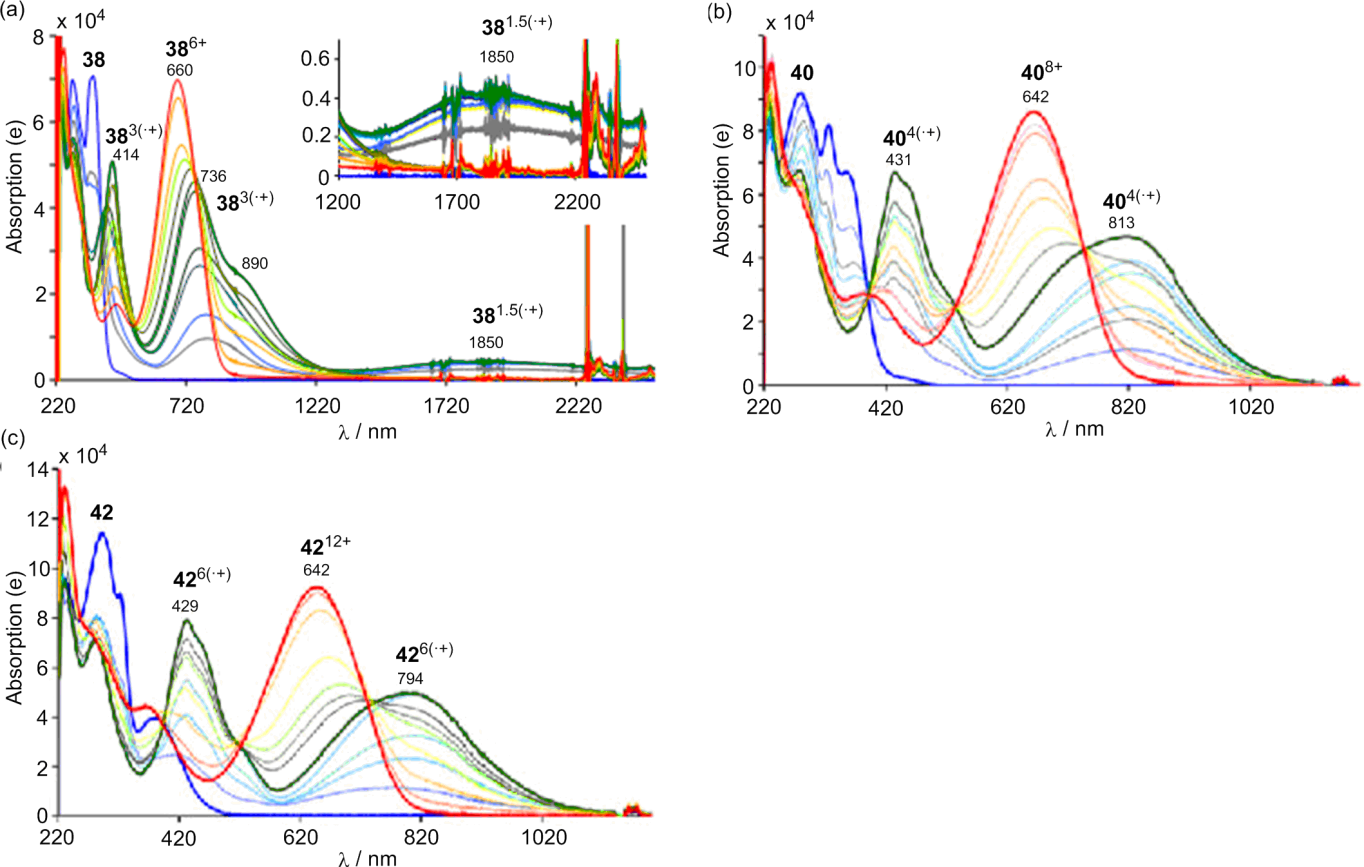
Stepwise oxidation of (a) **38** (0.02 mM), (b) **40** (0.05 mM), and (c) **42** (0.03 mM) with incremental addition of Fe(ClO_4_)_3_ in a mixture of CH_2_Cl_2_–CH_3_CN (2:1, v/v) at 25 °C. The blue line indicates the neutral absorption spectra, the green line the multiple TTF radical cations **38**^3(•+)^, **40**^4(•+)^, and **42**^6(•+)^, and the red line the TTF dications **38**^6+^, **40**^8+^, and **42**^12+^. Adapted with permission from [23]. Copyright 2011 American Chemical Society.

As shown in Figure 15, the stepwise chemical oxidation of **38**, **40**, and **42** with Fe(ClO_4_)_3_ in CH_2_Cl_2_–CH_3_CN 2:1 exhibits the typical changes in the absorption spectra. The addition of Fe(ClO_4_)_3_ up to 1 equiv with respect to each of the TTF units causes new absorption maxima at longer wavelength region (blue to green spectra). For the oxidation of **40**, the changes show several isosbestic points, indicating that each TTF unit is oxidized from the neutral to the radical cation (TTF^•+^) in a stepwise manner (Figure 15b). On the other hand, for **38** and **42**, there are no isosbestic points (Figure 15a,c). For **38**, a new broad peak around 1850 nm (intermolecular CR absorption) appears in the presence of 1.5 equiv of Fe(ClO_4_)_3_, which is attributed to the formation of an intermolecular face-to-face mixed valence complex. These results are consistent with the peak splitting of the CV. Furthermore, CV analysis of **44** exhibited two reversible ten-electron redox waves corresponding to the formation of **44**^10+^ and **44**^20+^.

Trisubstituted **38** showed polymorphism and formed single crystals from CH_2_Cl_2_, whereas it produced a yellow fibrous material from CH_2_Cl_2_–hexane 1:4. X-ray diffractometry (XRD) exhibited that fiber **38** possesses a hexagonal columnar structure different from single crystals. Furthermore, the spin-coated film of **38** has an amorphous structure. Interestingly, doping of single crystals, hexagonal fiber, and amorphous film of **38** with iodine vapor produced black CT-complexes having different assembled structure. After doping, electric conductivity of single crystals was σ_rt_ = 1.8 × 10^−2^ S cm^−1^ and the fiber was 1.9 × 10^−2^ S cm^−1^, whereas the amorphous film was 2.5 × 10^−3^ S cm^−1^. The difference in the conductivity reflects the molecular level alignments. Other star-shaped oligomers **39–44** also formed nanostructures fibers, particles and film, and doping with iodine produced black complexes which exhibited electric conductivities of σ_rt_ = 2.7 × 10^−3^–2.4 × 10^−2^ S cm^−1^ in spite of the non-planarity of the molecular frame of **39–44**.

**Scheme 4:**
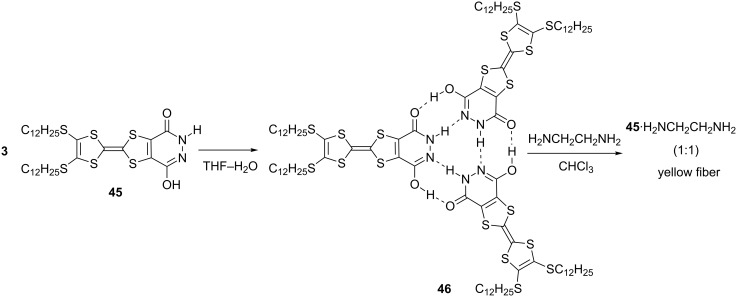
Pyridazine-3,6-diol-TTF **45** and its trimer **46**.

The pyridazine-3,6-diol-annulated TTF derivative **45** produced trimer **46** via hydrogen bonds in a THF–H_2_O solution (Scheme 4), in which micrometer-sized fibrous material was gradually formed [76]. The compressed pellet of the **46** fibers showed an electrical conductivity of σ_rt_ = 2.3 × 10^−4^ S cm^−1^ after doping with iodine vapor. The addition of ethylene diamine triggered the reorganization of the supramolecular structure **46**, and fine nanoscopic fibers composed of **45** and ethylene diamine (1:1) were produced from the CHCl_3_ solution. A compressed pellet of the fibers of **45**·H_2_NCH_2_CH_2_NH_2_ exhibited an electrical conductivity in the range of σ_rt_ = 1.5–10.0 × 10^−5^ S cm^−1^ after iodine doping.

Recently, conducting nanofibers derived from the self-assembly of TTF-appended dipeptides were reported [77]. Conductivity measurements performed on the nanofibers of TTF-appended dipeptides indicate a remarkable enhancement in the conductivity after doping with TCNQ (σ_rt_ = 1 × 10^−5^ S cm^−1^).

### Conducting nanostructures prepared from cation radicals

**Figure 16:**
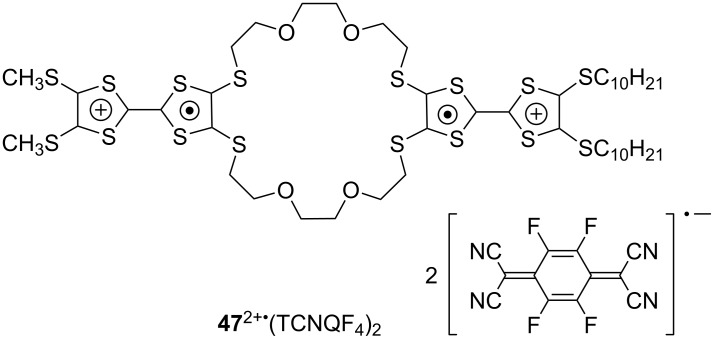
CT-complex of **47** with TCNQF_4_.

Molecular conductors derived from CT complexes and radical salts of TTFs are widely known [1], and mixed-valence (TTF_2_)*^n^*^+^ (0 < *n* < 1) was reported to form self-accembled conducting nanofibers (σ_rt_ = ~10^−2^ S cm^−1^) [78–82]. However, there is only a limited number of examples of nanofibers and nanorods prepared from CT complexes and radical salts of star-shaped and radially expanded TTF oligomers. One typical example is the conducting CT complex **47**^2+^·(TCNQF_4_^•−^)_2_ of amphiphilic TTF **47** and TCNQF_4_ (Figure 16) [83–84]. The fiber structure with typical dimensions of 2.5 nm (height) × 50 nm (width) × 1 μm (length) was constructed on a mica substrate by using the Langmuir–Blodgett (LB) technique, and the conductivity of the film composed the **47**^2+^·(TCNQF4^•−^)_2_ fiber was found to be on the order of σ_rt_ = 10^−3^ S cm^−1^.

**Figure 17:**
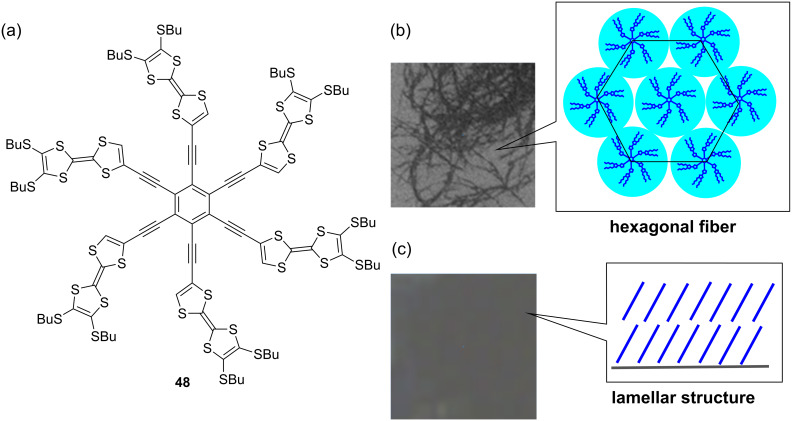
(a) Star-shaped TTF hexamer **48**. (b) Optical image of **48** fiber with a hexagonal structure. (c) Optical image of **48** film with a lamellar structure. Adapted with permission from [22]. Copyright 2007 American Chemical Society.

The stacking behavior of TTF in solution and in the solid state was employed as a driving force to construct higher aggregates by using the star-shaped hexakis(tetrathiafulvalenylethynyl)benzene **48** (Figure 17a). The TTF-hexamer **48** was synthesized by Sonogashira coupling of **21** with hexaiodobenzene (52%) [22]. As expected, **48** strongly self-aggregates in CHCl_3_ (*K*_a_ = 2.1 × 10^4^ M^–1^, 23 °C) and in other common organic solvents. To construct nanoobjects, a CHCl_3_ solution of **48** was diluted with hexane to afford dark blue fibers with a slim and curled fiber structure (40–90 nm wide, 30–100 nm thick and more than 10 μm long) (Figure 17b). On the other hand, a dark blue film was formed by casting a solution of **48** on a glass surface (Figure 17c). XRD studies on the fiber and the film of **48** revealed that the fiber has a hexagonal alignment, whereas the film has a lamellar structure with lateral order and π···π stacking. It is worth noting that the film of **48** prepared by casting a 0.1 wt % solution of **48** in CHCl_3_ exhibited a low carrier mobility of μ = 3 × 10^−6^ cm^2^ V^−1^ s^−1^, indicating a lamellar structure vertical to the substrate surface.

Oxidation of **48** with 1 and 3 equiv of Fe(ClO_4_)_3_ produced the analytically pure monocation **48**^•+^ClO_4_^−^ and trication **48**^3+^(ClO_4_^−^)_3_, respectively. The cationic species **48**^•+^ClO_4_^−^ and **48**^3+^(ClO_4_^−^)_3_ strongly self-aggregate in CHCl_3_ (*K*_a_ = 2.3–2.5 × 10^6^ M^–1^) and rather weakly aggregate in THF. Interestingly, in THF, **48**^•+^ClO_4_^−^ and **48**^3+^(ClO_4_^−^)_3_ exhibited the formation of stacked cylindrical structures with a radius of 11 Å and a height of 14–16 Å by small-angle X-ray scattering (SAXS). ESR spectra of **48**^•+^ and **48**^3+^ in CHCl_3_ at 23 °C showed 100% of spin for **48**^•+^ and 33% of spin for **48**^3+^. Therefore, the spin–spin interaction in **48**^•+^ is weak, whereas the spin–spin interaction in **48**^3+^ is strong.

The monocation **48**^•+^ClO_4_^−^ easily formed a hexagonal fiber from CHCl_3_–hexane solution, probably owing to the strong aggregation properties and molecular shape of the stacked **48**^•+^ClO_4_^−^, whereas trication **48**^3+^(ClO_4_^−^)_3_ produced a nanoparticle having a low internal regularity, presumably owing to the strong intermolecular TTF^•+^–TTF^•+^ interaction of **48**^3+^. A cast film of **48**^•+^ClO_4_^−^ shows a lamellar structure vertical to the substrate in a similar manner to the neutral **48** (Figure 17c). Interestingly, the structural difference between nanofiber and film of **48**^•+^ClO_4_^−^ leads to the different electric conductivities of wires (σ_rt_ = 1.1 × 10^−3^ S cm^−1^) and film (σ_rt_ = 3.1 × 10^-5^ S cm^−1^) depending on their stacking structures.

Cation radicals of pyrrole-fused TTF trimer **38** also formed conducting nanostructures when a CH_2_Cl_2_ solution of **38**^1.5(•+)^ was mixed with an excess amount of hexane. The XRD pattern of the fiber **38**^1.5(•+)^ is composed of a lamellar structure different from the neutral **38** fiber and an exhibited electric conductivity of σ_rt_ = 2.9 × 10^−4^ S cm^−1^. The lower conductivity of the fiber **38**^1.5(•+)^ as compared to the doped **38** fiber (σ_rt_ = 1.9 × 10^−2^ S cm^−1^) may be due to the difference in their internal structures.

## Conclusion

The construction of nanoobjects based on the self-assembly of TTFs were rapidly advanced, and a large number of functional properties such as electronic, magnetic, and optical properties were recently reported. Based on these developments of nanoscience, the construction of conducting nanoobjects has also been investigated to realize electrochemically-driven conformational control, redox-controlled gelation processes, redox switches, and molecular sensors. Furthermore, semiconductive fibers and rods of TTFs can be utilized for nanosized electric wires and wirings in nanoelectronics. The next key innovation in TTF-based nanoobjects is the fulfillment of nanofiber and nanorod with metallic conductivity and superconductivity. To achieve a high electric conductivity, further knowledge is necessary to fabricate a closely stacked ionic state with unfilled bands. If these innovative systems can be implemented, conducting nanoobjects find functions in a variety of mass use devices.

## Supporting Information

File 1 Determination of association constants (*K*_2_) of **23** by NMR and cyclic voltammetry analysis of **23**.
