# Supplementary material for: Star-shaped tetrathiafulvalene oligomers towards the construction of conducting supramolecular assembly
Source: Beilstein J Org Chem. 2015 Sep 10;11:1596–613. doi: 10.3762/bjoc.11.175 (PMC4660952; doi:10.3762/bjoc.11.175)
Supplement: File 1 — Determination of association constants (K2) of 23 by NMR and cyclic voltammetry analysis of 23. [file Beilstein_J_Org_Chem-11-1596-s001.pdf]

Supporting Information
for

Star-shaped tetrathiafulvalene oligomers towards the construction of conducting supramolecular assembly

Masahiko Iyoda[1*] and Masashi Hasegawa[2]

Address: [1]Department of Chemistry, Graduate School of Science and Engineering, Tokyo Metropolitan University, Hachioji, Tokyo 192-0397, Japan and [2]Department of Chemistry, School of Science, Kitasato University, 1-15-1 Kitasato, Minami-ku, Sagamihara, Kanagawa 252-0373, Japan

Email: MasahikoIyoda - iyoda@tmu.ac.jp

*Corresponding author

Determination of association constants (K_2) of 23 by NMR and cyclic voltammetry analysis of 23

Contents

S1. Determination of association constant by NMR technique S2
S2. Chemical shifts of H_a and association constants (K_2) of **23** in $CDCl_3$ (Table S1) S3
S3. Chemical shifts of H_a and association constants (K_2) of **23** in $CDCl_3$–CD_3CN 3:7 (Table S2) S4
S4. Cyclic voltammetry analysis of **23** in dilute and concentrated CH_2Cl_2 solutions S5

S1. Determination of association constant by NMR technique

The association constant of **23** was determined by ^{1}H NMR titration experiments. As mentioned in the text, the protons at the central benzene ring clearly shifted to higher field with increasing concentration. Thus, the central H_a (Scheme S1) varied from δ 7.478 to 7.457 as the concentration changed from 0.48 to 48.3 mM in $CDCl_3$ at 293 K. On the contrary, the chemical shift of H_a in 14.3 mM of **23** varied from δ 7.464 to 7.484 downfield as the temperature changed from 40 to −20 °C.

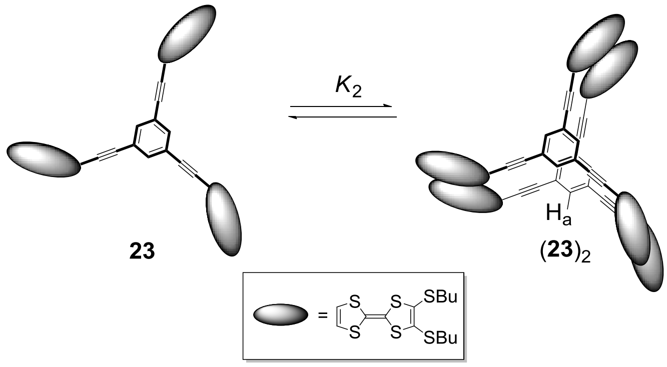

Scheme S1: Self-association of **23**

The association constants (K_2) of **23** were determined by assuming that monomer–dimer equilibrium is the predominant process. The chemical shifts of **23** were plotted against the concentrations and the data were treated by least-square curve-fitting to eq. 2, to determine K_2, δ_m, and δ_d, where δ_m and δ_d are chemical shifts of the monomer and dimer, and C is the total stoichiometric concentration.

$$K_2 = \frac{[(23)_2]}{[23]^2} \qquad \text{eq. 1}$$

$$\delta = \delta_m + (\delta_d - \delta_m)\left(1 + \frac{1 - \sqrt{8K_2C + 1}}{4K_2C}\right) \qquad \text{eq. 2}$$

The chemical shifts of the H_a proton, which show reasonable concentration and temperature dependence, were applied to the equations. The dilution curve and plotted points are shown in Figures S1 and S2, and the calculated parameters, K_2, δ_m, and δ_d, are summarized in Tables 1 and 2.

S2. Chemical shifts of H_a and association constants (K_2) of 23 in $CDCl_3$.

Table S1: Chemical shifts of H_a and calculated δ_d, δ_m, and K_2 in $CDCl_3$.

Conc	−10 °C	0 °C	10 °C	20 °C	30 °C	40 °C
48.3	7.4616	7.4604	7.4586	7.4567	7.4543	7.4518
32.2	7.4695	7.4677	7.4653	7.4634	7.4598	7.4567
21.5	7.4769	7.4744	7.4708	7.4677	7.4647	7.4610
14.3	7.4811	7.4781	7.4750	7.4714	7.4677	7.4640
9.5	7.4848	7.4811	7.4775	7.4738	7.4701	7.4659
4.8	7.4884	7.4848	7.4799	7.4762	7.4720	7.4683
0.95	7.4921	7.4878	7.4830	7.4787	7.4738	7.4695
0.48	7.4921	-	7.4830	7.4781	7.4744	7.4695
δ_d	7.304 ± 0.026	7.2948 ± 0.033	7.291 ± 0.035	7.291 ± 0.028	7.283 ± 0.051	7.278 ± 0.061
δ_m	7.493 ± 0.000	7.488 ± 0.000	7.4835 ± 0.000	7.479 ± 0.000	7.475 ± 0.000	7.470 ± 0.000
K_2	2.46 ± 0.47	2.05 ± 0.47	1.78 ± 0.43	1.58 ± 0.30	1.38 ± 0.45	1.22 ± 0.47

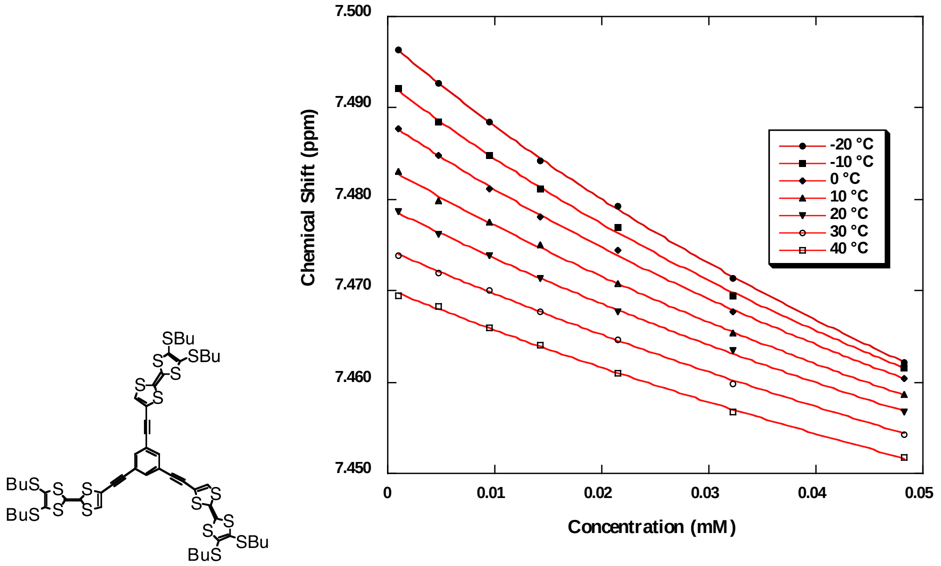

Figure S1: Curve fitting for H_a proton of **23** in $CDCl_3$ solution.

S3. Chemical shifts of H_a and association constants (K_2) of 23 in $CDCl_3$–CD_3CN (3:7)

Table S2: Chemical shifts of H_a and calculated δ_d, δ_m, and K_2 in $CDCl_3$–CD_3CN (3:7).

Conc. (mM)	−30 °C	−20 °C	−10 °C	0 °C	10 °C	20 °C	30 °C
10.00	–	7.4161	7.4381	7.4522	7.4626	7.4699	7.4729
8.44	7.4039	7.4320	7.4534	7.4656	7.4717	7.4766	–
6.49	7.4284	7.4510	7.4668	7.4760	7.4821	7.4845	7.4858
5.00	7.4461	7.4656	7.4778	7.4851	7.4888	7.4906	7.4909
4.33	7.4601	7.4760	7.4858	7.4913	7.4937	7.4943	7.4931
2.79	7.4802	7.4937	7.4979	7.5004	7.5004	7.5004	7.4992
2.50	7.4858	7.4968	7.5016	7.5029	7.5047	7.5022	7.4992
1.85	7.5029	7.5084	7.5102	7.5102	7.5084	7.5053	7.5022
1.39	7.5114	7.5145	7.5151	7.5132	7.5108	7.5077	7.5041
0.923	7.5218	7.5212	7.5193	7.5169	7.5139	7.5102	7.5065
0.723	7.5267	7.5248	7.5224	7.5187	7.5151	7.5114	–
0.462	7.5315	7.5285	7.5254	7.5211	7.5169	7.5130	7.5077
δ_d	6.9021 ± 0.075	6.9137 ± 0.062	6.9238 ± 0.107	6.9599 ± 0.179	6.9743 ± 0.154	6.9815 ± 0.089	6.9846 ± 0.208
δ_m	7.5445 ± 0.002	7.5370 ± 0.001	7.5310 ± 0.001	7.5253 ± 0.001	7.5203 ± 0.001	7.5151 ± 0.000	7.5100 ± 0.000
K_2	21.1 ± 3.85	14.5 ± 2.10	10.4 ± 2.46	7.98 ± 3.24	6.40 ± 2.21	5.01 ± 0.98	4.04 ± 1.84

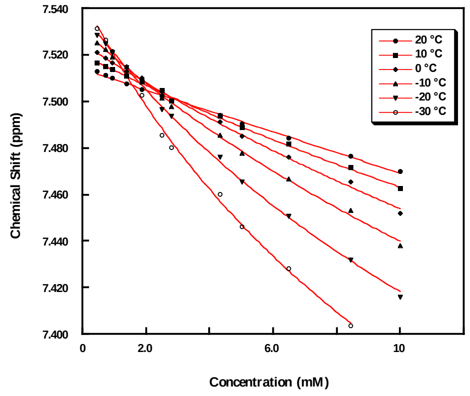

Figure S2: Curve fitting for H_a proton of **23** in CD_3CN–$CDCl_3$ solution.

S3. Cyclic voltammetry analysis of 23 in dilute and concentrated CH_2Cl_2 solutions.

The redox potentials of **23** were measured by cyclic voltammetry using 0.1 M *n*-Bu_4NClO_4, Pt as working electrode, Ag/Ag^+ as reference electrode, and Pt wire as counter electrode at 100 mV/sec. Potentials were referred to Fc/Fc^+. As shown in Figure S2, CV analysis of **23** in a dilute CH_2Cl_2 solution (1.9×10^{-5} M) showed two three-electron redox waves at 0.05 and 0.40 V vs Fc/Fc^+ corresponding to the formation of $\mathbf{23}^{3+}$ and $\mathbf{23}^{6+}$, whereas a similar CV analysis of **23** in a concentrated CH_2Cl_2 solution (1.2×10^{-3} M) displayed three reversible waves at –0.04, 0.14, and 0.47 V vs Fc/Fc^+ corresponding to the formation of $(\mathbf{23})_2^{3+}$, $(\mathbf{23})_2^{6+}$, and $(\mathbf{23})_2^{12+}$

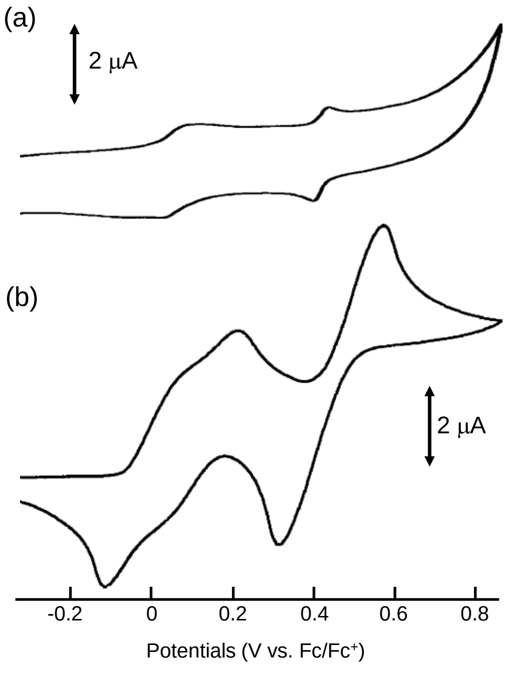

Figure S2: Cyclic voltammograms of **23**. (a) Dilute ($c = 1.9 \times 10^{-5}$ M) solution in CH_2Cl_2. (b) Concentrated ($c = 1.2 \times 10^{-3}$ M) solution in CH_2Cl_2.
